# Lowly Expressed Toxin Transcripts in Poorly Characterized Myanmar Russell’s Viper Venom Gland

**DOI:** 10.3390/biotech14040096

**Published:** 2025-12-04

**Authors:** Khin Than Yee, Jason Macrander, Olga Vasieva, Ponlapat Rojnuckarin

**Affiliations:** 1 Experimental Medicine Research Division, Department of Medical Research, Yangon 11191, Myanmar; 2 Biology Department, Florida Southern College, Lakeland, FL 33801, USA; 3 Institute of Integrative Biology, University of Liverpool, Liverpool L69 7ZB, UK; ovasieva@ingenets.com; 4 Excellence Center in Translational Hematology, Department of Medicine, Faculty of Medicine, Chulalongkorn University, Bangkok 10330, Thailand

**Keywords:** Russell’s viper, venom gland, lowly expressed toxin transcript, therapeutic candidates

## Abstract

In Myanmar, Russell’s viper (*Daboia siamensis*) bite is a significant public health problem. In this study, we expend upon our previous RNA-sequencing approach to characterize candidate toxin genes encoding *D. siamensis* toxins. The mRNA was extracted from Myanmar Russell’s viper venom glands. The RNAseq was performed using Illumina next-generation sequencing. Subsequently, candidate toxin transcripts were recognized by the Venomix pipeline. This study focused on 29 unique cDNA sequences representing eight newly identified venom gene families with low-to-moderate expression levels. These transcripts represented 0.088% of the total number of transcripts in the dataset. The translated protein sequences were analyzed for their conserved motifs and domains to predict their functions. They were neprilysins (bioactive peptide inactivators), cystatins (protease inhibitors with anti-metastatic activities), waprin and vipericidin (antimicrobial peptides), veficolin (platelet and complement activation), vespryns and three-finger toxins (elapid toxin homologs causing neurotoxic activity and tissue damage), and endothelial lipases (unknown function). Their functional activities should be further investigated for potential therapeutic applications, for example, in cancer or antibiotic-resistant infections.

## 1. Introduction

Russell’s viper (*Daboia siamensis*) is a widespread species found in South and Southeast Asia. In Myanmar, it is responsible for 8000–10,000 venomous snake bites from 2016 to 2019, with 5% of those resulting in mortalities [1]. More recent reports estimate mortality rate as high as 8–10% [2,3]. Hemostatic and renal abnormalities are common in patients bitten by Myanmar Russell’s viper, and the most common cause of death are coagulopathy and acute kidney injury [2,3]. The venom composition of *D. siamensis* varies significantly across its geographic range and is further influenced by factors such as age, sex, diet, season, and frequency of venom extraction [4,5,6,7,8]. Their venom consists primarily of protein-based toxins that work synergistically to immobilize, kill, and begin the digestion of prey. In humans, these proteins often cause severe hemostatic and renal disturbances, which are among the leading causes of death following envenomation by *D. siamensis* in Myanmar [2,3].

The clinical manifestations of envenomation are characterized by the toxin components in the venom [9]. Snake venom contains a diverse array of peptides, which display diverse biochemical and pharmaceutical properties [10]. Moreover, toxin families exhibit synergistic interactions that amplify systemic and local envenomation. For example, the combined action of snake venom metalloproteinases (SVMPs) and phospholipases (PLA_2_) showed amplification of individual toxin effects and enhanced the overall toxicity of *D. russelli* venom [11]. In addition, a minor amount of hyaluronidase was shown to intensify the overall toxicity of *Naja naja* venom [12]. Thus, it is worthwhile to study even lowly expressed toxins, as some may behave synergistically.

The complexity of venom is better explored using a combination of omics technologies such as genomics, transcriptomics, and proteomics [13]. Among these, transcriptomics offers insight into venom content and its predicted physiological and biochemical roles. Additionally, this approach facilitates the identification of transcript domains, infers evolutionary patterns, and quantification and characterization of venom composition and function [14]. An integrated multi-omics study of *Daboia siamensis* from Thailand found numerous previously unreported venom proteins [15]. Only two deduced toxin sequences, *D. siamensis* apoptosis-inducing protein (DSAIP) and waprin, were analyzed for structure–function prediction in that study. Myanmar and Thailand DSAIP showed divergence in amino acids in the sequence alignment.

Comparative venom gland transcriptomics of snakes from different countries elucidated geographical venom variation and novel sequences, which are important for regional next-generation antivenom and novel therapeutic development [15,16]. In addition to biomedical application, transcriptomic studies are applicable to evolutionary studies. A comparative transcriptomic study involving various tissues from *Bothrops jararaca* demonstrated the complex process of recruitment and production of venom in snakes [17]. Mechanisms surrounding venom evolution occurred at multiple genome levels under strong selection revealed in an integrated genomic, transcriptomic, and proteomic study of the king cobra [18].

In our previous study, we used comparative transcriptomics to characterize the venom composition from four adult *D. siamensis* species. Putative venom transcripts were classified into 23 distinct toxin gene families, yielding 53 unique full-length transcripts [8]. Although we focused on the most abundantly expressed toxins, i.e., C-type lectins (CTLs), Kunitz-type serine protease inhibitors, disintegrins, and bradykinin-potentiating peptide/C-type natriuretic peptides (BPP-CNP) precursors, the rare components and lowly expressed components of their venoms remained overlooked despite having incredible medical potential. For example, lowly expressed protease inhibitors used in self-preservation have the potential to be antivenom therapy or as inhibitors of protease-mediated processes like tumor metastasis in humans [19,20]. Other constitutes are antimicrobial peptides that may be employed by snakes to fight against microbes present in the prey they consume. These peptides might have capabilities to become clinical treatments for drug-resistant organisms in the future [21]. Finally, various peptides in the elapid toxin families have been found in vipers as residuals of evolution [22]. The activities of these proteins in vipers remain to be investigated.

## 2. Materials and Methods

Venom glands were dissected from four adult *D. siamensis* specimens (two adult males and two adult females) provided by a local Myanmar snake farm. The venom was extracted four days before gland dissection to stimulate toxin transcription. The venom glands were removed under anesthesia, and the tissues were then cut into 5 × 5 mm pieces and kept in RNAlater solution at −80 °C. Total RNA was extracted using a Total RNA Purification Kit (Jena Bioscience GmbH, Jena, Germany). Then, the mRNA was isolated using a FastTrack MAG mRNA Isolation Kit (Invitrogen, Carlsbad, CA, USA) and sequenced on the Illumina HiSeq platform at Macrogen Inc. Geumchun-gu, Seoul, Republic of Korea.

The quality of the raw data from the Illumina platform was checked with FastQC (version 0.11.9) and cleaned up using Trimmomatric (0.32). The transcriptomes were then assembled de novo using the Trinity software version 2.13.2 [23]. Candidate toxin genes were identified using the Venomix pipeline, and their expression levels were calculated in transcripts per million (TPM) using RSEM software version 1.33 [24]. The annotation of generated transcript sequences was aligned by standalone BLASTP (v. 2.10.0) to the UniProtKB/Swiss-Prot. The identified toxin candidates were aligned with previously described venom proteins using Clustal Omega Online from EMBL-EBI [25]. The transcripts from this study were named as “c” (component/contig), “g” (subgraph/trinity gene), and “i” (path sequence/trinity isoform) followed by numbers. The letter “F” stood for female, “M” for male, “PL” for partial-length, and “FL” for full-length transcripts. Within each toxin group, the number of mature toxins with signal regions were determined using SignalP [26], as well as the number of unique nucleotide sequence ORFs.

## 3. Results and Discussion

### 3.1. Classification and Expression Profile of Venom Gland Transcripts

In this study, we further explored the lowly expressed transcripts and characterized their potential functions and biomedical applications. Among the preciously overlooked candidate venom transcripts, we were able to classify 53 unique transcripts into 23 toxin gene families representing eight protein families: neprilysin (2), cystatin (5), waprin (1), vipericidin (1), veficolin (2), endothelial lipase (9), vespryn (ohanin) (8), and three-finger toxin (1). The expression levels of these transcripts were found to be moderate to low (TPM = 1.49 to 213.37). The majority of the toxin candidates resembled toxins originally isolated from elapids, which usually exhibit neurological toxicities and local tissue damage. Some toxin transcripts were predicted to display antimicrobial activity and anti-metastatic effects (Table 1).

**Figure 1:**
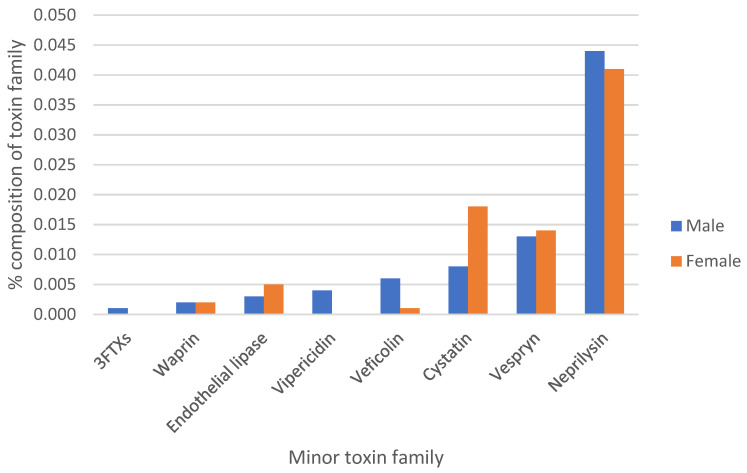
Comparison of minor toxin transcript compositions (the lowly expressed toxin families) of male and female snakes.

The expression levels of these minor toxin transcripts were also found to be varied between males and females, as seen in major toxin transcripts (Table S1). Three-finger toxin and vipericidin transcripts were only identified in male transcriptomes. In Figure 1, the cystatin, endothelial lipase, and vespryn transcripts were more highly expressed in female than male samples, while neprilysin and veficolin transcripts were more highly expressed in male than female snakes. This result showed that both major and minor toxin transcripts were sex-specifically expressed in their venom glands. In *Daboia palaestinae* (Palestine viper) species, a highly similar abundance of venom transcripts across the left and right venom glands of an individual snake was seen, indicating no partitioning in venom production [27]. The expression levels related to individual and condition-specific factors still need to be examined in this species.

### 3.2. Neprilysins

**Figure 2:**
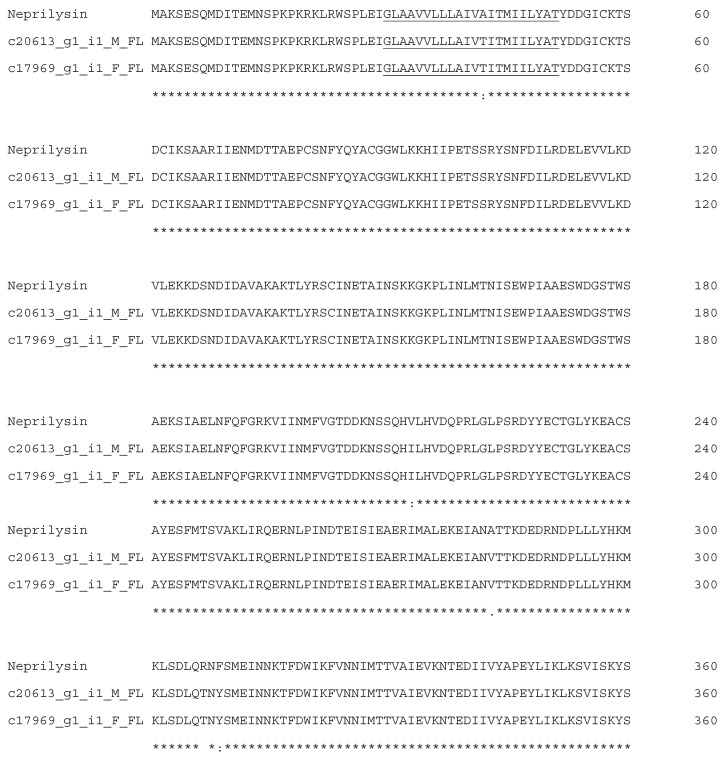

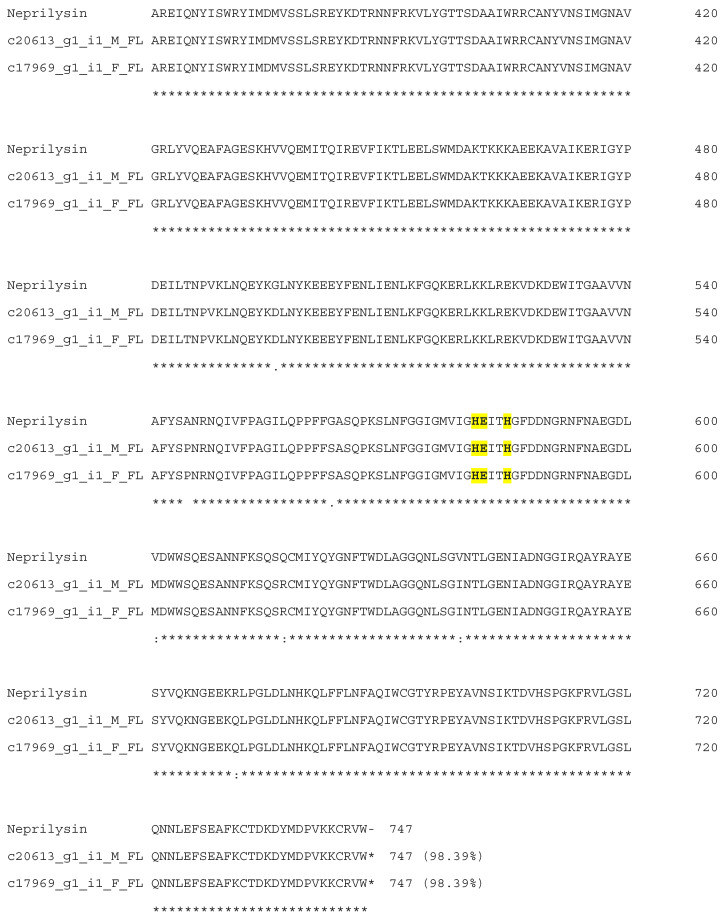
Alignment of Neprilysin 1 (QHR82779.1) sequences from Anatolian meadow viper (Vipera anatolica senliki) with deduced sequences of Myanmar Russell’s viper neprilysin annotated transcripts (c20613_g1_i1_M_FL and c17969_g1_i1_F_FL) from the Myanmar Russell’s viper transcriptomes. The predicted transmembrane domain was underlined. The conserved zinc-binding (HExxH) motif are in bold and highlighted in yellow. (*) fully conserved residues; (:) strongly similar residues; and (.) weakly similar residues.

Neprilysins (NEPs) are zinc metalloendopeptidase of approximately 750 amino acids (~110 kDa) having an active site with the HExxH motif [28]. Our analysis of the Myanmar Russell’s viper transcriptomes revealed two full-length predicted neprilysin transcripts with TPM values of 64.38 and 213.37, sharing 98.39% identity with Neprilysin 1 (QHR82779.1) from the Anatolian meadow viper (*Vipera anatolica senliki*) (Figure 2). NEPs are synthesized as integral membrane thermolysin-like proteins but can released into the extracellular space. In humans, NEPs digest natriuretic peptide hormones, providing a new perspective for NEP inhibitors and their application towards the clinical treatment of heart failure [29]. Although their functions in snake envenoming have not been elucidated, they are found in the venoms of *Echis pyramidum leakeyi*, *Ophiophagus hannah*, and *Naja kaouthia* [16,30,31]. It has been suggested that NEPs from the spider *Avicularia juruensis* are synergistic, as they degrade the extracellular matrix, facilitating access of other toxins and aiding prey digestion [32]. NEPs interact with a variety of peptides, such as natriuretic, vasodilatory, and neuropeptides, suggesting potential roles in neurological and circulatory toxicities [30].

### 3.3. Snake Venom Cystatins

**Figure 3:**
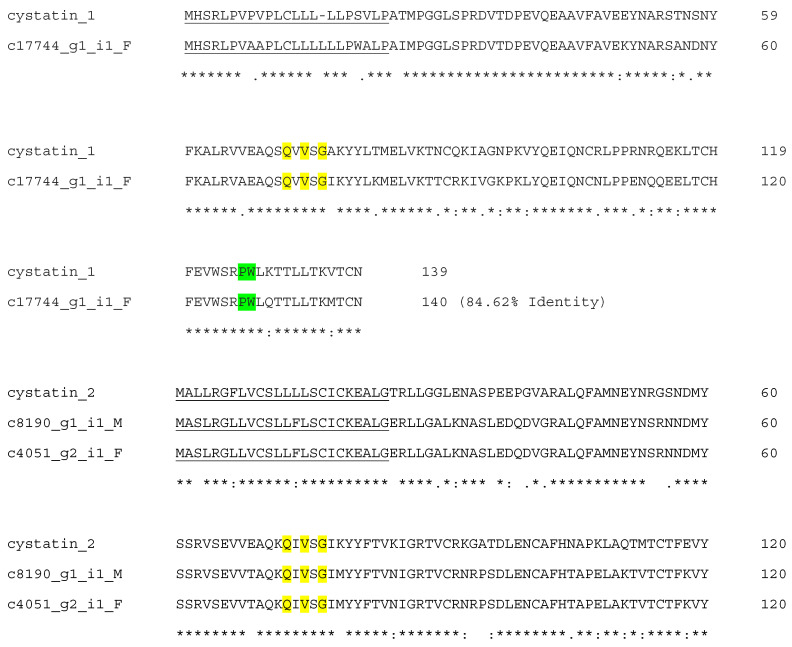

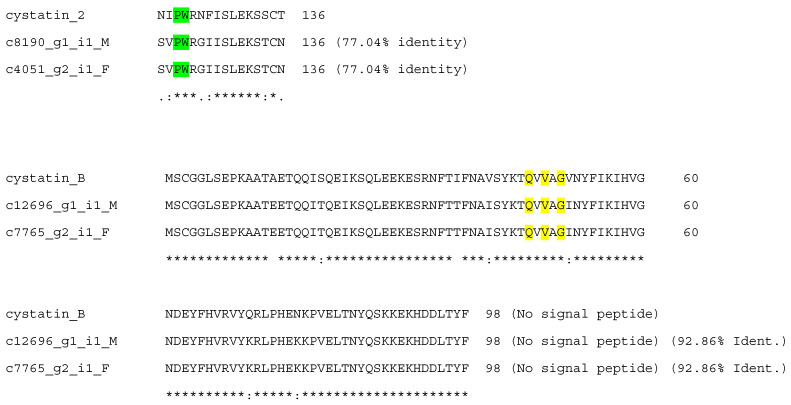
Alignment of deduced amino acid sequences of Myanmar Russell’s viper cystatin homologs with Cystatin-1 (XP_015672096.1) from *Protobothrops mucrosquamatus*; Cystatin-2 (J3SE80.1) from *Crotalus adamanteus*; and Cystatin-B (XP_015667611.1) from *Protobothrops mucrosquamatus*. The signal peptide is underlined. The conserved QxVxG and PW motif were highlighted in yellow and green, respectively. (*) fully conserved residues; (:) strongly similar residues; and (.) weakly similar residues.

Snake venom cystatin (sv-cystatin) are cysteine protease inhibitors belonging to subfamily 2 of the cystatin family. They have 120 amino acid residues with two disulfide bonds [33]. The sv-cystatin isolated from *Naja naja atra* exhibits a shorter sequence than other type-2 cystatins [20] and has an anti-metastatic effect on tumor cells [34]. Our analysis recovered a total of five putative transcripts across three isoforms, specifically cystatin-1 (Type-1 cystatin), cystatin-2 (Type-2 cystatin), and cystatin-B (Type-1 cystatin) (Table 2). The conserved QXVXG region was identified in all three isoforms and the PW motif in two of the isoforms (Figure 3). Both Type-1 and Type-2 cystatins are key regulators of various cancer cells associated with cathepsins and affect tumor cell invasion and metastasis [35]. These conserved regions are essential for the protease inhibitory action of cystatin [36]. Type-2 cystatin isolated from *N. naja atra*, containing the QxVxG motif, showed anti-invasion and anti-metastasis effects in a lung-metastasis mouse model through the reduction in proteinases activity and Epithelial–Mesenchymal Transition (EMT) [37]. Its recombinant sv-cystatin protein also suppressed mouse melanoma invasion, metastasis, and growth in vitro and in vivo [38], and therefore has potential pharmaceutical applications as an antiangiogenic and anti-metastatic therapeutic agent [39].

### 3.4. Waprins

**Figure 4:**
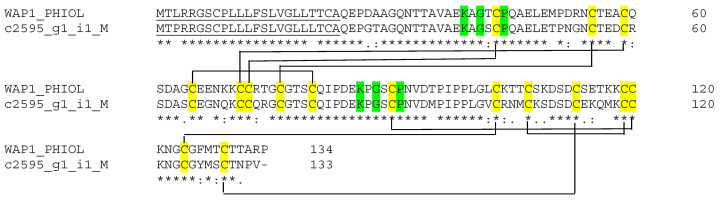
Sequence alignment of putative waprin from Myanmar Russell’s viper transcriptome with Waprin-Phil (A7X4K1) from *Philodryas olfersii*. The signal peptide is underlined. The cysteine residues are highlighted in yellow. Identical residues are marked in green. The lines connecting corresponding cysteine pairs show the disulfide-bonding pattern. (*) fully conserved residues; (:) strongly similar residues; and (.) weakly similar residues.

Waprins possess WAP (whey acidic proteins) domains, each consisting of 50 amino acid residues that contain eight conserved cysteine residues forming four disulfide bonds [33,40]. Waprin transcripts encoding one or two WAP domain proteins have been reported in elapid and colubrid snake venoms [41]. Proteins containing a WAP domain have diverse functions. For example, omwaprin isolated from *Oxyuranus microlepidotus* lyses bacteria membrane without hemolytic activity on human erythrocytes [42], suggesting potential antimicrobial properties. We recovered a single full-length hypothetical transcript with a TPM of 10.34 and 77.86% sequence identity to WAP1 (A7X4K1) from *Philodryas olfersii*. There were two WAP domains with a four-disulfide core structure (Figure 4). This disulfide-bond-constrained tertiary structure is essential for the antibacterial function of the protein by the localization of important positively charged functional residues on the surface of the protein [43].

### 3.5. Cathelicidins

**Figure 5:**
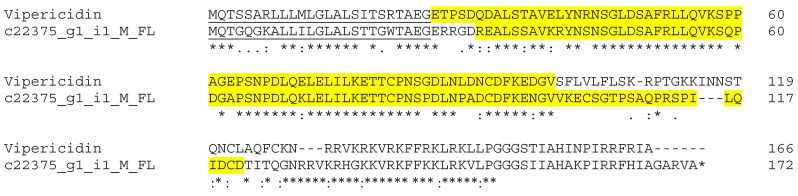
Sequence alignment of putative CRAMP transcript from Myanmar Russell’s viper transcriptomes with CAMP Vipericidin (A0A6P9AP78) from *P. guttatus*. The Cathelicidin domains are highlighted in yellow. Signal peptide is underlined. (*) fully conserved residues; (:) strongly similar residues; and (.) weakly similar residues.

Cathelicidins are one of the main families of antimicrobial peptides (AMPs), with an important role in host defense based on their broad antimicrobial activity. The primary structure of cathelicidins contains a conserved N-terminus a signal peptide and a cathepsin L inhibitor (cathelin) domain precedeing a C-terminal region. Cathelicidins possess amphipathic hydrophilic–hydrophobic helices that can disrupt microbial membranes [44]. Snake venom cathelicidin-related antimicrobial peptides (CRAMPs) have been identified from three Asian elapids and four South American pit vipers (Table 3) [45]. Our transcriptome analysis identified one full-length predicted CRAMP transcript with 64.12% identity to the cathelicidin-related peptide Vipericidin (A0A6P9AP78) from *Pantherophis guttatus* (Figure 5). Cathelicidin-BF purified from *Bungarus fasciatus* venom efficiently kills bacteria and some fungi, as well as antibiotic-resistant microorganisms [46]. A cathelicidin-derived peptide from king cobra (OH-CATH30) exerts antimicrobial activity against a multitude of virulent bacteria from human sources and has potential for treating various bacterial infections [47]. The hypothetical vipericidin from Myanmar Russell’s viper should be further studied for its potential antimicrobial properties.

### 3.6. Veficolins

**Figure 6:**
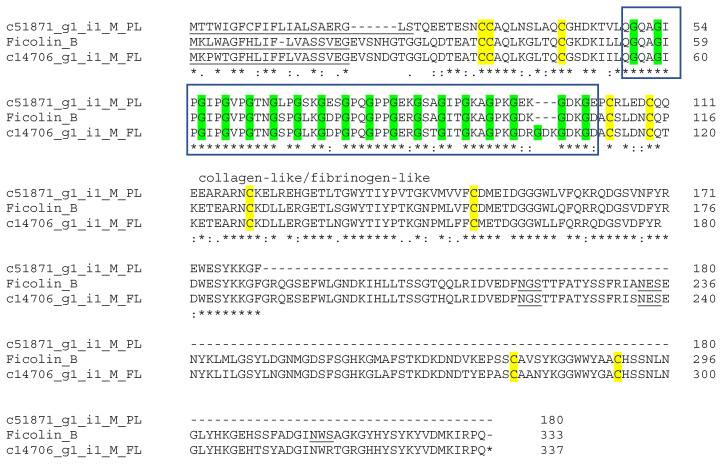
The putative veficolin toxin sequences from Myanmar Russell’s viper transcriptomes. The signal peptides are underlined. The amino acid glycines are in green color. The G-X-Y collagen-like repeats are included in the boxes. The conserved cysteines are highlighted in yellow. The putative N-linked glycosylation sites (N-X-S/T) are underlined. (*) fully conserved residues; (:) strongly similar residues; and (.) weakly similar residues.

Veficolins (venom ficolin) are structurally similar to ficolins, which are mammalian proteins with collagen-like and fibrinogen-like domains [48]. Veficolins (ryncolin 1 and ryncolin 2) have been identified in *Ceberus rynchops* (dog-faced water snake). They may induce platelet aggregation and/or complement activation [49]. Two predicted veficolin transcripts (one partial and one full-length) were identified, sharing 68.65% and 90.50% sequence identity to Ficolin B (A0A098LYG5) from *Pantherophis guttatus* (Figure 6). Their biological activities need further evaluation to determine whether their role during envenomation involves platelet consumption and/or immune activation.

### 3.7. Endothelial Lipases

Endothelial lipases (ELs) are members of the triglyceride lipase family and synthesized by endothelial cells [50]. Among lipases, they have the typical Ser–His–Asp catalytic triad and a very short lid, as well as a deletion of a loop (the β9 loop) in contrast with pancreatic lipases [51,52]. ELs display primarily phospholipase A_1_ (PLA_1_) activity [49,50], hydrolyze triglycerides and phosphatidylcholine at the Sn-1 position [53], and play an essential role in regulating lipid metabolism and inflammatory responses in humans [54]. We identified nine full-length hypothetical EL transcripts with 98.86 to 99.37% sequence identity to EL proteins from *Vipera analotica senliki* (A0A6G5ZW01) [55] (Figure S1). Lipases are rarely identified in snake venom [56], probably due to their low expression among other toxins, and their functional roles should be further investigated.

### 3.8. Vespryns

Venom PRY-SPRY domain-containing proteins (Vespryns) is a new family of venom proteins, comprising ohanin from *Ophiophagus hannah*. Ohanin was purified from crude *Ophiophagus hannah* (king cobra) venom. It has a molecular weight of 12 kDa with 107 amino acid residues and a unique single cysteine residue. Ohnanin induces hypolocomotion and hypernociception in mice [57]. Other members of this protein family have been identified as Thai cobrin from *Naja kaouthia* and ohanin-like proteins from *Naja naja atra*, *Lachesis muta*, and *Tropidechis carintus* [58]. From the current transcriptomes, we identified eight full-length putative transcripts containing PRY-SPRY domain, six from male transcriptomes and two from female transcriptomes. Their length varied from 286 to 549 amino acids with TPM values of 2.25 to 12.14. Two putative transcripts have three conserved LDP, WEVE, and LDYE motifs of the B30.2-like domain, while others showed variations in amino acids at those regions (Table 4). Their B30.2-like domains shared 31–35% sequence similarity with the ohanin precursor (AAR07992.2) from *O. hannah* (Figure S2). Ohanin and ohanin-like proteins are the shortest members of the B30.2 family, and their conserved motifs are responsible for their main activities [59]. Moreover, the length and distinctive N-terminal domains are also responsible for their various activities [22]. So, the currently identified putative vespryns with different lengths and various functional motifs remain to be further studied for their activities.

### 3.9. Three-Finger Toxins

**Figure 7:**
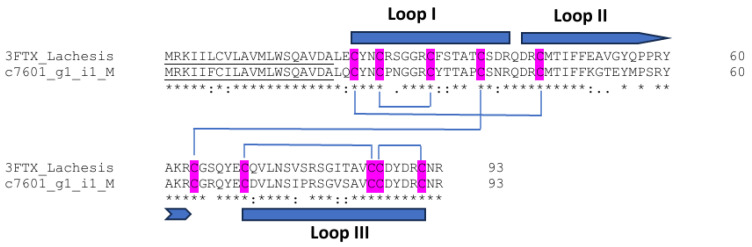
Sequence alignment of full-length hypothetical 3FTXs transcript from Myanmar Russell’s viper transcriptomes with respective homologs from *Lachesis muta* (ABD52883). The leader sequences are underlined. Cysteine residues are highlighted in pink. (*) fully conserved residues; (:) strongly similar residues; and (.) weakly similar residues.

Three-finger toxins (3FTXs) contain nonenzymatic polypeptides with 60–74 amino acid residues. They exhibit three β-stranded loops extending from a small, globular hydrophobic core that contains four conserved disulfide bridges (short-chain neurotoxins). They are generally monomers, and their various functions are due to the robust and highly versatile three-finger protein scaffold [60]. The positively charged residues located at the tip of loop II in non-conventional 3FTXs recognize various respective cholinergic receptors [61]. Candoxin, a non-conventional 3FTX from the Malayan krait (*Bungarus candidus*) (*Elapidae*) venom, produces reversible neuromuscular blockage at presynaptic nicotinic acetylcholine receptors (nAChRs) [62]. Denmotoxin, a 3FTX from the colubrid snake *Boiga dendrophilia*, displays potent irreversible postsynaptic neuromuscular activity with bird-specificity [63]. 3FTXs are the main venom components of elapid snakes [61,64], whereas 3FTX mRNAs and corresponding proteins of viperid snake venoms are poorly characterized [65]. Among these, a short-chain neurotoxin-like protein was isolated from Indian Russell’s viper (*Daboia russelli russelli*) (Viperidae) (DNTx I) with postsynaptic neurotoxic and cytotoxic properties [66]. From the current transcriptomes, one full-length hypothetical 3FTX transcript was identified containing 73 amino acids with five possible disulfide bridges (Figure 7) and 77.42% identity to 3FTX from *Lachesis muta* (ABD52883). To date, 3FTXs have only been identified at the transcript level within two viperid species, TFT-AF from *Azemiops feae* and VN-TFT from *Vipera nikolskii*, functioning as antagonists of nAChRs of neuronal- and muscle-type [67]. Among elapids, 3FTXs such as mambalgins from *Dendroaspis polylepis* and the cobra cardiotoxin CTX-I from *Naja kaouthia* serve as prototypes for new therapeutic agents for pain and type 2 diabetes, respectively [64]. The finding of 3FTXs at the transcript level in Viperidae indicates their minor contribution to venom composition compared with elapids. This may be due to 3FTX genes in Viperidae snakes evolving under the constraints of negative selection pressure, resulting in ancillary rather than major role in envenomation [68]. The biological functions of the currently deduced non-conventional 3FTX from Myanmar Russell’s viper transcriptomes deserve further investigation.

Our previous venom gland transcriptome profile showed the abundance of C-type lectins (CTLs) (26%), Kunitz-type protease inhibitors (KSPIs) (19%), disintegrins (18%), and bradykinin-potentiating peptide/C-type natriuretic precursors (BPP-CNPs) (16%), PLA_2_ (5.5%), and serine proteases (SVSPs) (5%) [8]. In a proteomic study, six venom protein families were identified from the Myanmar *D. siamensis* venom: serine proteinases, metalloproteinases, PLA_2_, LAAOs, VEGF, and C-type lectin-like proteins. The discrepancy between the snake venom gland transcriptome and proteome has also been reported in *D. palaestinae* [27], *Vipera ammodytes ammodytes* [69], *Bothropoides pauloensis* [70], and *Echis ocellatus* [71]. These findings suggest that venom composition is influenced by complex transcriptional and translational mechanisms and indicate importance of combining transcriptomic and proteomic approaches to understand the precise composition of venom proteins.

Different types of minor toxin transcripts were identified in transcriptomes of different snake species. In *D. palaestinae*, LAAOs, disintegrin, VEGF, Kuntiz, PLB, hyaluronidase, PDE, 5′-NT, natriuretic peptide, Kazal-type serine proteases inhibitor, CRISPs, cobra venom (CVPF), neprilysin, waprin, and NGF constituted < 10% of the transcriptomes [27]. In *Vipera ammodytes ammodytes*, serine protease inhibitors, VEGF, CRISPs, LAAOs, and venom nerve growth factors (VNGFs) accounted for <5% of the transcriptome [69]. The transcripts encoding aspartic proteinases, snake venom growth factors (VEGF and NGF), and hyaluronidases attributed for 4.5%, 2.9%, and 0.3% of the *Echis ocellatus* transcriptome, respectively [71]. The interaction of lowly expressed toxins transcripts with the expression of major toxin transcripts in venom production should be studied further.

Proteomic studies of Russell’s viper venom have demonstrated variation in abundant toxin families across geographical regions [72], supporting the variation in hemotoxic clinical features [73]. Envenoming by Russell’s vipers is characterized by local edema, tissue necrosis, hemorrhage, coagulopathy, and nephrotoxicity. C-type lectins act on platelets, contributing to thrombus formation [74]. The Kunitz-type serine protease inhibitors in *Daboia* species exhibit anticoagulant effect [75]. Disintegrins act on specific integrin receptors of platelets and extracellular matrix proteins, probably contributing to bleeding pathology [76]. Bradykinin-potentiating peptides and natriuretic peptides released from BPP-CNP transcripts may play a role in hypotension during snake envenomation.

PLA_2_ from Myanmar Russell’s viper showed neurotoxic, myotoxic, cytotoxic, edema-inducing, and indirect hemolytic activities [77]. SVMPs, especially RVV-X, activate factor X in the coagulation cascade, leading to formation of thrombin and fibrin clots [78]. Moreover, SVSPs play an important role in blood coagulation system, particularly RVV-V, which specifically acts on factor V to activate blood coagulation [79]. The most hemotoxic effects of the Myanmar Russell’s viper among other regional Russell’s viper may be contributed by the different isoforms within toxin groups and different compositions of toxin families in the venom acting individually as well as synergistically.

Synergism between different toxins and toxin complexes gives significant toxicity to the snake venom. The predominant toxins, PLA_2_, SVMPs, SVSPs, and 3FTXs, play essential roles in synergistic processes [80]. The toxin synergy of two major families in *D. russelii* venom, PLA_2_ and SVMPs, results in more cytotoxic and hemorrhagic effects than either toxin group alone [11].

The quantitatively minor venom component hyaluronidase potentiates toxicity of crotoxin from *Crotalus durissus terrificus* venom [81]. Similarly, some minor toxin groups participate synergistically in the deleterious effects of major toxin groups in *D. siamensis* species. L-amino acid oxidase and PDE, together with PLA_2_ and SVMPs, alter renal function by promoting platelet-activating factor in the isolated perfused rabbit kidney (IPK) model [82]. The aforementioned four toxin groups synergistically induce renal tubular toxicity by increasing oxidative stress production and elevating inflammatory cytokines in the same IPK model [83]. The roles of lowly transcribed toxins in venom toxicity should be further investigated.

## 4. Conclusions

This study was a continuation of our previous work [8], allowing us to expand our investigation of Myanmar Russell’s viper venom diversity by more extensively exploring toxin candidates expressed at low levels. The expression profiles of both major and minor transcripts are sex-specific. Putative protease inhibitors, antimicrobial peptides, and proteins of unknown functions with elapid toxin scaffolds were newly identified from the Myanmar Russell’s viper transcriptomes. This study highlights the importance of diving deep into comparative transcriptomics studies for a thorough analysis of venom gland composition and for the discovery of new venom components with potential pharmaceutical and other applications. The present results make an important contribution toward elucidating the structures of individual toxins and pave the way for new therapeutic and/or biotechnological applications for snake venoms.

## Figures and Tables

**Table 1:** Rare and newly found toxin genes in Myanmar Russell’s viper transcriptome.

Family	Predicted Function	No. of Sequences/No. of Mature/ No. of Unique ORF	Amino Acid	Domain Structure	Conserved Motifs
Neprilysin	Vasoactive peptide inactivation	2/2/1	750	Zinc Metalloendopeptidases and N-terminal Transmembrane Domain	HExxH active site
Cystatin	Protease inhibition Anti-metastatic effect	5/5/3	175	Cysteine Protease Inhibitor	QxVxG and PW
Waprin	Antibacterial activity Protease inhibition	1/1/1	133	Two Whey Acid Protein (WEP) Domains	Eight disulfide bonds KxGxCP motif
Vipericidin	Antimicrobial activity	1/1/1	172	Cathelicidin (Antimicrobial Peptide) Domain	Amphiphile peptide causing membrane disruption
Veficolin	Platelet aggregation Complement activation	2/1/2	337	Collagen-like Domain Fibrinogen-like Domain	Gxx collagen-like repeat
Vespryn (Ohanin)	Neurotoxicity	8/0/7	256–549	PRY-SPRY Domain	LPD, WEVE, and LDYE motif
Three-finger toxins	Neurotoxicity Tissue damage	1/1/1	93	Three Loops Extending From The Core	Five disulfide bridges for central core stabilization
Endothelial lipases	Unknown	9/1/4	316–493	Triglyceride lipase	S-(GxSxG)-H-D catalytic triad RxDxxD Ca^2+^ binding

**Table 2:** Transcripts with annotation of cystatins.

No	Contig ID	Annotation	Accession No.	Species	Amino Acid Length	TPM Value
1	c4051_g2_i1_F_FL	cystatin-2	J3SE80.1	Crotalus adamanteus	137	137
2	c8190_g1_i1_M_FL	cystatin-2	J3SE80.1	Crotalus adamanteus	137	19.77
3	c17744_g1_i1_F_FL	cystatin-1	XP_015672096.1	Protobothrops mucrosquamatus	175	8.99
4	c12696_g1_i1_M_FL	cystatin-A	XP_015667611.1	Protobothrops mucrosquamatus	98	113.67
5	c7765_g2_i1_F_FL	cystatin-A	XP_015667611.1	Protobothrops mucrosquamatus	98	36.79

**Table 3:** Snake venom cathelicidin-related antimicrobial peptides from some snake species.

Antimicrobial Peptides	Species	Common Name	Accession No.
NA-CATH	Naja atra	Chinese cobra	B6S2X0
OH-CATH	Ophiophagus hannah	King cobra	B6S2X2
BF-CATH	Bungarus fasciatus	Banded krait	B6D434
Pt-CRAMP1	Pseudonaja textilis	Eastern brown snake	U5KJJ1
Pt-CRAMP2	Pseudonaja textilis	Eastern brown snake	U5KJM6
Crotalicidin (Ctn)	Crotalus durissus terrificus	Eastern brown snake	U5KJM4
Bastroxicidin (BatxC)	Bothrops atrox	South American pit viper	U5KJC9
Lutzicidin	Bothrops lutzi	South American pit viper	U5KJT7

**Table 4:** Amino acid residues variation in three conserved motif regions of the vespryn deduced amino acids of Myanmar Russell’s viper transcriptome.

No.	Contig ID	Amino Acid Length	LDP Motif	WEVE Motif	LDYE Motif	TPM
1.	c18829_g1_i1_M_FL	549	LDP	WEVE	LDYE	12.14
2.	c11270_g2_i1_F_FL	493	LDP	WEVE	LDYE	5.70
3.	c18865_g3_i1_M_FL	255	LDP	WELE	LDYV	9.30
4.	c19046_g2_i2_M_FL	516	IDG	WQVF	LDYK	2.32
5.	c10539_g2_i1_F_FL	516	IDG	WQVF	LDYK	2.25
6.	c17242_g1_i1_M_FL	286	LDP	WVVE	LEF	5.78
7.	c17242_g1_i2_M_FL	302	LDP	WVVE	LKH	4.66
8.	c20069_g2_i1_M_FL	482	LDP	WEVT	LSF	6.05

## Data Availability

The original contributions presented in this study are included in the article/Supplementary Material. Further inquiries can be directed to the corresponding authors.

## Supplementary Materials

The following supporting information can be downloaded at https://www.mdpi.com/article/10.3390/biotech14040096/s1; Table S1: Expression profile of toxin groups between male and female Russell’s viper venom glands; Figure S1: Sequence alignment of putative endothelial lipases from Myanmar Russell’s viper transcriptomes and that from *V. a. senliki* (A0A6G5ZW01). The signal peptide is underlined. The catalytic serine (GxSxG motif) of the active site is highlighted in green. The aspartic acid (D) and histidine (H) residues are in blue and Ca^2+^ binding sites in yellow. (*) fully conserved residues; (:) strongly similar residues; and (.) weakly similar residues; Figure S2: Sequence alignment of B30.2-like domains of putative Vesprys from Myanmar Russell’s viper transcriptomes and that of Pro-ohanin (AAR07992.2) from *O. hannah*. The LDP motif, WEVW motif, and LDYE motif are highlighted in green, yellow, and pink, respectively. (*) fully conserved residues; (:) strongly similar residues; and (.) weakly similar residues.
