# Supplementary material for: Lowly Expressed Toxin Transcripts in Poorly Characterized Myanmar Russell’s Viper Venom Gland
Source: BioTech (Basel). 2025 Dec 4;14(4):96. doi: 10.3390/biotech14040096 (PMC12730704; doi:10.3390/biotech14040096)
Supplement: Supplementary file 1 [file biotech-14-00096-s001.zip › biotech-3906748-supplementary.pdf]

Article

Lowly Expressed Toxin Transcripts in Poorly Characterized Myanmar Russell's Viper Venom Gland

Supplementary Materials

Supplemental Figure S1. Sequence alignment of putative endothelial lipases from Myanmar Russell's viper transcriptomes and that from *V. a. senliki* (A0A6G5ZW01). The signal peptide is underlined. The catalytic serine (GxSxG motif) of the active site is highlighted in green. The aspartic acid (D) and histidine (H) residues are in blue and Ca^{2+} binding sites in yellow. (*) fully conserved residues; (:) strongly similar residues; (.) weakly similar residues.

```
VIPAN_Lipase          --------------MLQGN-----SNTVLLLLAAFRTLPMLGLCFFICFVAYCARAGQEC  41
c19855_g2_i11_M_FL    MCSLFLKISSEGAPMISR--------------------------------------GQEC  22
c19855_g2_i4_M_FL     ---------------------------------------MLGLCFFICFVAYCARAGQEC  21
c11312_g1_i2_F_FL     ------------------------------------------------------------  0
c11312_g1_i3_F_FL     MCSLFLKISSEGAPMISRAERVTSDTDLSRYTRPSHATFTAKLHSSSCH-INKSITGQEC  59
c11312_g1_i4_F_FL     ------------------------------------------------------------  0
c11312_g1_i5_F_FL     ------------------------------------------------------------  0
c19855_g2_i2_M_FL     ------------------------------------------------------------  0
c19855_g2_i5_M_FL     ------------------------------------------------------------  0
c19855_g2_i12_M_FL    MCSLFLKISSEGAPMISRAERVTSDTDLSRYTRPSHATFTAKLHSSSCH-INKSITGQEC  59


VIPAN_Lipase          PKFTDLNFGNAVIGTDLKVQLLLYTRRNKECAELLDEHNIATSTHFNVTKNIVVIIHGYR  101
c19855_g2_i11_M_FL    PKFTDLNFGNAVIGTDLKVQLLLYTRRNKECAELLDEHNIATSTHFNITKNIVVIIHGYR  82
c19855_g2_i4_M_FL     PKFTDLNFGNAVIGTDLKVQLLLYTRRNKECAELLDEHNIATSTHFNITKNIVVIIHGYR  81
c11312_g1_i2_F_FL     ------------------------------------------------------------  0
c11312_g1_i3_F_FL     PKFTDLNFGNAVIGTDLKVQLLLYTRRNKECAELLDEHNIATSTHFNITKNIVVIIHGYR  119
c11312_g1_i4_F_FL     ------------------------------------------------------------  0
c11312_g1_i5_F_FL     ------------------------------------------------------------  0
c19855_g2_i2_M_FL     ------------------------------------------------------------  0
c19855_g2_i5_M_FL     ------------------------------------------------------------  0
c19855_g2_i12_M_FL    PKFTDLNFGNAVIGTDLKVQLLLYTRRNKECAELLDEHNIATSTHFNITKNIVVIIHGYR  119


VIPAN_Lipase          FTGSPPIWIDTIKNLLLEKQDFNIIIVDWNRGATTVNYFSAVASAKKVVPRLTHIIDQML  161
c19855_g2_i11_M_FL    FTGSPPIWIDRIKNLLLEKQDFNIIIVDWNRGATTVNYFSAVASAKKVVPRLTHLIDQML  142
c19855_g2_i4_M_FL     FTGSPPIWIDRIKNLLLEKQDFNIIIVDWNRGATTVNYFSAVASAKKVVPRLTHLIDQML  141
c11312_g1_i2_F_FL     ----------------------------------------------------------ML  2
c11312_g1_i3_F_FL     FTGSPPIWIDRIKNLLLEKQDFNIIIVDWNRGATTVNYFSAVASAKKVVPRLTHLIDQML  179
c11312_g1_i4_F_FL     ----------------------------------------------------------ML  2
c11312_g1_i5_F_FL     ----------------------------------------------------------ML  2
```

```
c19855_g2_i2_M_FL      ----------------------------------------------------------ML  2
c19855_g2_i5_M_FL      ----------------------------------------------------------ML  2
c19855_g2_i12_M_FL     FTGSPPIWIDRIKNLLLEKQDFNIIIVDWNRGATTVNYFSAVASAKKVVPRLTHLIDQML  179
                                                                                 **

VIPAN_Lipase           ENGVAVDSIYMIGVSLGAHIAGFVGKAYNGKIGRITGLDPAGPLFTRKLANERLDHTDAQ  221
c19855_g2_i11_M_FL     ENGVAVDSIYMIGVSLGAHIAGFVGKAYNGKIGRITGLDPAGPLFTRKLANERLDHTDAQ  202
c19855_g2_i4_M_FL      ENGVAVDSIYMIGVSLGAHIAGFVGKAYNGKIGRITGLDPAGPLFTRKLANERLDHTDAQ  201
c11312_g1_i2_F_FL      ENGVAVDSIYMIGVSLGAHIAGFVGKAYNGKIGRITGLDPAGPLFTRKLANERLDHTDAQ  62
c11312_g1_i3_F_FL      ENGVAVDSIYMIGVSLGAHIAGFVGKAYNGKIGRITGLDPAGPLFTRKLANERLDHTDAQ  239
c11312_g1_i4_F_FL      ENGVAVDSIYMIGVSLGAHIAGFVGKAYNGKIGRITGLDPAGPLFTRKLANERLDHTDAQ  62
c11312_g1_i5_F_FL      ENGVAVDSIYMIGVSLGAHIAGFVGKAYNGKIGRITGLDPAGPLFTRKLANERLDHTDAQ  62
c19855_g2_i2_M_FL      ENGVAVDSIYMIGVSLGAHIAGFVGKAYNGKIGRITGLDPAGPLFTRKLANERLDHTDAQ  62
c19855_g2_i5_M_FL      ENGVAVDSIYMIGVSLGAHIAGFVGKAYNGKIGRITGLDPAGPLFTRKLANERLDHTDAQ  62
c19855_g2_i12_M_FL     ENGVAVDSIYMIGVSLGAHIAGFVGKAYNGKIGRITGLDPAGPLFTRKLANERLDHTDAQ  239
                       ************************************************************

VIPAN_Lipase           FVDVIHTDTDGFGLQDPLGNIDFYPNGGTDQPGCPKTILSGSAYFKCDHQRSVFLYMSSL  281
c19855_g2_i11_M_FL     FVDVIHTDTDGFGLQDPLGNIDFYPNGGTDQPGCPKTILSGSAYFKCDHQRSVFLYMSSL  262
c19855_g2_i4_M_FL      FVDVIHTDTDGFGLQDPLGNIDFYPNGGTDQPGCPKTILSGSAYFKCDHQRSVFLYMSSL  261
c11312_g1_i2_F_FL      FVDVIHTDTDGFGLQDPLGNIDFYPNGGTDQPGCPKTILSGSAYFKCDHQRSVFLYMSSL  122
c11312_g1_i3_F_FL      FVDVIHTDTDGFGLQDPLGNIDFYPNGGTDQPGCPKTILSGSAYFKCDHQRSVFLYMSSL  299
c11312_g1_i4_F_FL      FVDVIHTDTDGFGLQDPLGNIDFYPNGGTDQPGCPKTILSGSAYFKCDHQRSVFLYMSSL  122
c11312_g1_i5_F_FL      FVDVIHTDTDGFGLQDPLGNIDFYPNGGTDQPGCPKTILSGSAYFKCDHQRSVFLYMSSL  122
c19855_g2_i2_M_FL      FVDVIHTDTDGFGLQDPLGNIDFYPNGGTDQPGCPKTILSGSAYFKCDHQRSVFLYMSSL  122
c19855_g2_i5_M_FL      FVDVIHTDTDGFGLQDPLGNIDFYPNGGTDQPGCPKTILSGSAYFKCDHQRSVFLYMSSL  122
c19855_g2_i12_M_FL     FVDVIHTDTDGFGLQDPLGNIDFYPNGGTDQPGCPKTILSGSAYFKCDHQRSVFLYMSSL  299
                       ************************************************************

VIPAN_Lipase           QHNCDITAYPCESYMDYRNGKCVSCNFKSLPCPVIGYYADKWKSYILEKNPPLTTAYFDT  341
c19855_g2_i11_M_FL     QHNCDITAYPCESYMDYRNGKCVSCNFKSLPCPVIGYYADKWKSYILEKNPPLTTAYFDT  322
c19855_g2_i4_M_FL      QHNCDITAYPCESYMDYRNGKCVSCNFKSLPCPVIGYYADKWKSYILEKNPPLTTAYFDT  321
c11312_g1_i2_F_FL      QHNCDITAYPCESYMDYRNGKCVSCNFKSLPCPVIGYYADKWKSYILEKNPPLTTAYFDT  182
c11312_g1_i3_F_FL      QHNCDITAYPCESYMDYRNGKCVSCNFKSLPCPVIGYYADKWKSYILEKNPPLTTAYFDT  359
c11312_g1_i4_F_FL      QHNCDITAYPCESYMDYRNGKCVSCNFKSLPCPVIGYYADKWKSYILEKNPPLTTAYFDT  182
c11312_g1_i5_F_FL      QHNCDITAYPCESYMDYRNGKCVSCNFKSLPCPVIGYYADKWKSYILEKNPPLTTAYFDT  182
c19855_g2_i2_M_FL      QHNCDITAYPCESYMDYRNGKCVSCNFKSLPCPVIGYYADKWKSYILEKNPPLTTAYFDT  182
c19855_g2_i5_M_FL      QHNCDITAYPCESYMDYRNGKCVSCNFKSLPCPVIGYYADKWKSYILEKNPPLTTAYFDT  182
c19855_g2_i12_M_FL     QHNCDITAYPCESYMDYRNGKCVSCNFKSLPCPVIGYYADKWKSYILEKNPPLTTAYFDT  359
                       ************************************************************

VIPAN_Lipase           SDEDPFCMYHYSLDIITWNKSTRRGFINIKITDNSGNTIESRINSDAAVFQQYRQAKILA  401
```

```
c19855_g2_i11_M_FL      SDEDPFCMYHYSLDIITWNKSTRRGFINIKITDNSGNTIESRINSDAAVFQQYRQAKILA  382
c19855_g2_i4_M_FL       SDEDPFCMYHYSLDIITWNKSTRRGFINIKITDNSGNTIESRINSDAAVFQQYRQAKILA  381
c11312_g1_i2_F_FL       SDEDPFCMYHYSLDIITWNKSTRRGFINIKITDNSGNTIESRINSDAAVFQQYRQAKILA  242
c11312_g1_i3_F_FL       SDEDPFCMYHYSLDIITWNKSTRRGFINIKITDNSGNTIESRINSDAAVFQQYRQAKILA  419
c11312_g1_i4_F_FL       SDEDPFCMYHYSLDIITWNKSTRRGFINIKITDNSGNTIESRINSDAAVFQQYRQAKILA  242
c11312_g1_i5_F_FL       SDEDPFCMYHYSLDIITWNKSTRRGFINIKITDNSGNTIESRINSDAAVFQQYRQAKILA  242
c19855_g2_i2_M_FL       SDEDPFCMYHYSLDIITWNKSTRRGFINIKITDNSGNTIESRINSDAAVFQQYRQAKILA  242
c19855_g2_i5_M_FL       SDEDPFCMYHYSLDIITWNKSTRRGFINIKITDNSGNTIESRINSDAAVFQQYRQAKILA  242
c19855_g2_i12_M_FL      SDEDPFCMYHYSLDIITWNKSTRRGFINIKITDNSGNTIESRINSDAAVFQQYRQAKILA  419
                        ************************************************************

VIPAN_Lipase            GFYLDFGNISTITLTFSTKSTVGPKYKLRVLEMRLKSLSHPERIQLCRYDFILVENVETT  461
c19855_g2_i11_M_FL      GFYLDFGNISTITLTFSTKSTVGPKYRLRVLEMKLKSLSHPERIQLCRYDFILVENVETT  442
c19855_g2_i4_M_FL       GFYLDFGNISTITLTFSTKSTVGPKYRLRVLEMKLKSLSHPERIQLCRYDFILVENVETT  441
c11312_g1_i2_F_FL       GFYLDFGNISTITLTFSTKSTVGPKYRLRVLEMKLKSLSHPERIQLCRYDFILVENVETT  302
c11312_g1_i3_F_FL       GFYLDFGNISTITLTFSTKSTVGPKYRLRVLEMKLKSLSHPERIQLCRYDFILVENVETT  479
c11312_g1_i4_F_FL       GFYLDFGNISTITLTFSTKSTVGPKYRLRVLEMKLKSLSHPERIQLCRYDFILVENVETT  302
c11312_g1_i5_F_FL       GFYLDFGNISTITLTFSTKSTVGPKYRLRVLEMKLKSLSHPERIQLCRYDFILVENVETT  302
c19855_g2_i2_M_FL       GFYLDFGNISTITLTFSTKSTVGPKYRLRVLEMKLKSLSHPERIQLCRYDFILVENVETT  302
c19855_g2_i5_M_FL       GFYLDFGNISTITLTFSTKSTVGPKYRLRVLEMKLKSLSHPERIQLCRYDFILVENVETT  302
c19855_g2_i12_M_FL      GFYLDFGNISTITLTFSTKSTVGPKYRLRVLEMKLKSLSHPERIQLCRYDFILVENVETT  479
                        **************************:******:*************************

VIPAN_Lipase            FTPIPCYEINMQDN-         475
c19855_g2_i11_M_FL      FTPIPCYEINMQDN*         456 (99.86%)
c19855_g2_i4_M_FL       FTPIPCYEINMQDN*         455 (98.90%)
c11312_g1_i2_F_FL       FTPIPCYEINMQDN*         316 (99.37%)
c11312_g1_i3_F_FL       FTPIPCYEINMQDN*         493 (98.86%)
c11312_g1_i4_F_FL       FTPIPCYEINMQDN*         316 (99.37%)
c11312_g1_i5_F_FL       FTPIPCYEINMQDN*         316 (99.37%)
c19855_g2_i2_M_FL       FTPIPCYEINMQDN*         316 (99.37%)
c19855_g2_i5_M_FL       FTPIPCYEINMQDN*         316 (99.37%)
c19855_g2_i12_M_FL      FTPIPCYEINMQDN*         493 (98.86%)
                        **************
```

Supplemental Figure S2. Sequence alignment of B30.2-like domains of putative Vesprys from Myanmar Russell's viper transcriptomes and that of Pro-ohanin (AAR07992.2) from *O. hannah*. The LDP motif, WEVW motif and LDYE motif are highlighted in green, yellow and pink, respectively. (*) fully conserved residues; (:) strongly similar residues; (.) weakly similar residues.

```
c10539_g2_i1_F_FL     KVYITIDGSTAHPWLCCQGT-------VLSLANQNQNVPDLPGRFDQEWCALGCGGFSAG   53
c19046_g2_i2_M_FL     KVYITIDGSTAHPWLCCQGT-------VLSLANQNQNVPDLPGRFDQEWCALGCGGFSAG   53
c18865_g3_i1_M_FL     KGISTLDPDFSSALLPLDQRNAGDRESQHRLP-ALHYVPDTPQRLDSRALVLGCDGFTSG   59
Pro_ohanin            KADVTFDSNTAFESLVVSPD----KKTVENVGV-PKGVPDSPERFSSSPCVLGSPGFRSG   55
c20069_g2_i1_M_FL     LENLTLDPATAQANLVVSED----GKRVECVEHKQAVSLDDPQRFDKSNCLVSRQSFSQG   56
c11270_g2_i1_F_FL     TGDLTLDPETAHPNLLLSED----RKSVKFVEQRLRDLPDSPKRFTIYPCVLATEGFVSG   56
c18829_g1_i1_M_FL     TGDLTLDPETAHPNLLLSED----RKSVKFVEQRLRDLPDSPKRFTIYPCVLATEGFVSG   56
c17242_g1_i1_M_FL     KVEVTLDPMTAFPTLIISED----LKSVYMGEQ-AQDLPNNPERFKYFPCVLGTEGINSG   55
c17242_g1_i2_M_FL     KVEVILDPKTAYPALILSED----QKEVHMGEQ-TQVLFDNPERFRFLPGVLGAEGIDSG   55
                       :*   :  *  .                 : * *:     :.  .:  *

c10539_g2_i1_F_FL     WHCWQVFVQEAGNAPVRGRACWALGVALESVCRKGSLQLSPQEGIWAVGKSVEGETVAFS   113
c19046_g2_i2_M_FL     WHCWQVFVQEAGNAPVRGRACWALGVALESVCRKGSLQLSPQEGIWAVGKSVEGETVAFS   113
c18865_g3_i1_M_FL     RYFWELEVGD--------GEFWAVGVTRDPSKKKGMMDFSPEEGIWAVGLWK-GQHWALT   110
Pro_ohanin            KHFFEVKYGT--------QREWAVGLAGKSVKRKGYLRLVPEERIWQKGLWW-LRRLE--   104
c20069_g2_i1_M_FL     EHYWEVTVDD--------KPRWALGLISAETGRKGRLHATPSNGFWLVGCKE-GKSYEAY   107
c11270_g2_i1_F_FL     RHYWEVEVGE--------KTHWALGVCKDSVSRKGETTSVPETGYWRVRLLN-GEKYAAT   107
c18829_g1_i1_M_FL     RHYWEVEVGE--------KTHWALGVCKDSVSRKGETTSVPETGYWRVRLLN-GEKYAAT   107
c17242_g1_i1_M_FL     TSEWVVEVGR--------AKQWAIGAVRESIEREGYLNIMANEGFWVLQLMN-GE-YEIS   105
c17242_g1_i2_M_FL     TLEWVVEVGK--------AKEWAVGIARESVERKYSEDITITQGFWVLQLAQ-GE-YQVS   105
                      : :             **:*      ::         *      .

c10539_g2_i1_F_FL     K---VHKKLSLQRPLRNLQVRLDYKAEEVEFLDAETG---ASLYTFRTGAFLGEKACPFF   167
c19046_g2_i2_M_FL     K---VHKKLSLQRPLRNLQVRLDYKAEEVEFLDAETG---ASLYTFRTGAFLGEKACPFF   167
c18865_g3_i1_M_FL     S---PVTALSLSRHPQRIRVSLDYVGECVTFTDADTE---DPIFTFPPASFNGTRAHPLL   164
Pro_ohanin            -----TDSDKLQKGSGKIIVFLDYDEGKVIFDLDGEVTTI------QA-NFNGEEVVPFY   152
c20069_g2_i1_M_FL     VEHKEPRSLKLEGKPNRIGIYLSFDDGLLAFYDASDEDNLVQIFAFHE-RF-TGTVYPFF   165
c11270_g2_i1_F_FL     TT--PFTPLHITIKPKRVGVFLDYEAGKLSFYNVTDR---SHMYTFSD-TF-TEKLWPFF   160
c18829_g1_i1_M_FL     TT--PFTPLHITIKPKRVGVFLDYEAGKLSFYNVTDR---SHMYTFSD-TF-TEKLWPFF   160
c17242_g1_i1_M_FL     TT--PKTILPLWKTIQRILVSLEFSLGKLSFYDVDSM---EIIYTFNY-PF-FEKMFPFF   158
c17242_g1_i2_M_FL     TS--PPIKLTLWKSPQRILIILKHDYDRLIFYNADSM---DHIFTFNY-PF-SEKVFPFV   158
                           :     .: : *..    : *                *      *:
```

Identities (%)

```
c10539_g2_i1_F_FL      YLGQAGVTLQ--------------CEVYKPPTSLYYR*    190   33  (33)

c19046_g2_i2_M_FL      YLGQAGVTLQ--------------CEVYKPPTSLYYR*    190   33  (33)

c18865_g3_i1_M_FL      WVVGFPAQTVFLRRMGVKYPVGVHEMDIMSP*------    195   35  (35)

Pro_ohanin             YIGAR-VSLA-----------NL---------------    163  100 (100)

c20069_g2_i1_M_FL      DVCWH-DKGK-----------NSHPLIIYTPESQER*-    189   32  (32)

c11270_g2_i1_F_FL      YPGIR-AGRK-----------NAAPLVLRPPTDWE*--    183   35  (35)

c18829_g1_i1_M_FL      YPGIR-AGRK-----------NAAPLVLRPPTDWE*--    183   35  (35)

c17242_g1_i1_M_FL      LTWDK-----------------KNPLKISTDYSIR*--    176   33  (33)

c17242_g1_i2_M_FL      LVRDK-----------------ETPLKISPRSSV*---    175   31  (31)
```

Supplemental Table S1. Expression profile of toxin groups between male and female Russell's viper venom glands

**Toxin groups**		**Male**		**Female**	
**Major toxin groups**		**Total TPM**	%	**Total TPM**	%
1.	**CRISP**	4335.68	0.879	1313.36	0.790
2.	**LAAO**	7713.56	1.564	3205.92	1.929
3.	**SVSP (Proteases)**	12890.40	2.615	15067.87	9.068
4.	**Metalloproteinases**	18584.90	3.769	43450.10	26.146
5.	**VEGF**	18709.00	3.794	8132.29	4.890
6.	**PLA2**	25764.10	5.225	10661.55	6.416
7.	**Disintegrin**	76998.90	15.616	10198.59	6.137
8.	**BPP-CNPs**	78769.20	15.975	24184.75	14.553
9.	**KSPI**	110026.00	22.314	16861.07	10.146
10.	**C-type lectin**	136940.00	27.772	32369.63	19.479
**Minor toxin groups**					
1.	**Three-finger toxins**	3.47	0.001	0	0
2.	**Waprin**	10.34	0.002	2.77	0.002
3.	**Endothelial lipases**	13.74	0.003	7.87	0.005
4.	**Vipericidin**	18.33	0.004	0	0
5.	**Veficolin**	27.80	0.006	2.38	0.001
6.	**Cystatin**	40.65	0.008	29.22	0.018
7.	**Vespryn (Ohanin)**	62.74	0.013	23.67	0.014
8.	**Neprilysin**	218.51	0.044	68.71	0.041
